# Inflammation, Ulceration, and Induration of the Cervix Uteri

**Published:** 1845-09

**Authors:** 


*Inflammation, Ulceration, and Induration of the Cervix Uteri.*—Dr. J. H. Bennett states that when the inflammation and induration are the immediate or even proximate result of miscarriages, difficult labors, or of deep lacerations of the organ, there is frequently more or less inflammation of the uterus itself. If metritis is present, general antiphlogistic measures, such as bleeding from the arm, &c., may be indicated. So active a treatment, however, will seldom be found necessary. Generally speaking, the patients have been exhausted by flooding, by mucoso-purulent discharges, or by previous medication, and are not in a state to bear very energetic measures. Complete rest in bed, diluents, tepid hip-baths, emollient injections, poultices to the abdomen, and a few leeches to the hypogastric or iliac region, will nearly always subdue the general inflammation of the uterus in the course of a few days, so as to admit of examination with the speculum, which must not be attempted as long as the uterus itself is acutely inflamed. As soon, however, as the inflammatory symptoms have subsided, the examination should be made, as nothing will then so much tend completely to allay the irritation of the uterine system, as cauterisation of the ulcerated surface which, instead of exposing the patient to metritis, as has been asserted, seems to exercise the same beneficial effect over the surrounding uterine inflammation, that cauterization of an ulcer of the cornea exercises over ophthalmia. When the inflammation and induration affect the cervix only, general antiphlogistic treatment is scarcely ever required. Superficial cauterization, injections, rest, and light diet, will often cure the ulceration, and resolve the induration, the latter being generally occasioned and kept up by the former. This, however, occurs when the nutrition of the engorged cervix has not been deeply modified by the subacute inflammation of which it has been the seat perhaps for months or even years. Not unfrequently, especially in very chronic cases, the hypertrophy only diminishes to a certain extent under this treatment, and even that very slowly, and then remains stationary, whether the ulceration heal or not. The cervix uteri continues to be the seat of general chronic inflammatory induration, and while this is the case, the patient cannot be said to be cured, as she still suffers from the uterine prolapsus, sensation of pelvic heaviness, and bearing down, severe lumbar pains, constipation, vesical and rectal irritation, &c., and is liable to a relapse of the ulceration, with its attendant symptoms. As this chronic induration is exceedingly difficult of removal, the treatment must be directed from the commencement with the view to effect its cure. Complete rest is indispensable. In addition, if there be much hypogastric pain, large thin linseed poultices, frequently changed, should be applied to the part, and tepid hip-baths used twice a day. The cauterization of the ulcerated surface should be practised by the acid nitrate of mercury, or caustic potash. Emollient and astringent injections should also be thrown up. Attention must be paid at the same time to the bowels, and to the general health. If under this plan of treatment, the ulcer presents a more healthy appearance, and the hypertrophy of the neck appears rapidly to decrease, it may be continued, as it will probably prove quite sufficient to effect a cure. If this should not be the case, however, other remedies must be had recourse to, the most efficacious being the application of leeches directly to the cervix uteri. Their application is easily effected, and they are most certainly extremely useful in subduing deep-seated chronic inflammation in this region. The following is the easiest way to apply them; after introducing an ordinarily conical speculum, wipe off the mucus which covers the cervix with a little lint or sponage, and then place the leeches in the interior of the speculum. Over its external orifice place a bit of linen, which depress with the finger into the speculum. In the concavity thus formed, place some lint or cotton, and then with the forceps push the whole towards the uterine neck. The linen carries the leeches before it, and presses them against the os uteri. On pulling out the linen and the lint, with which the speculum was plugged, in the course of about ten minutes, it will nearly always be found that all the leeches have taken. They generally fill well in this situation, and the flow of blood is often considerable after they have fallen. Six, eight, ten, or twelve leeches may be applied at once, according to the effect to be produced, and they should be re-applied (query, fresh ones used?) several times, at intervals of five, six, eight, or ten days, when necessary, until the desired effect is produced. The leech-punctures always heal readily. Their bite is not felt by the patient, unless they fix on the vagina, which they cannot do if the speculum is properly introduced. The instrument must be held by the patient, or by the nurse, while the leeches are on. They generally fall off, but it is sometimes necessary to bring them away, after they have filled. If all these measures fail in causing resolution of the hypertrophied cervix, after trying the effects of time, and frictions with the hydriodate of potash and other solvents, the engorged cervix should be deeply cauterized either with the Vienna paste, or by the actual cautery. The eschar which forms in either case is much deeper than when the fluid caustics are used. The inflammation which accompanies its separation is also much more intense, and generally propagates itself to the entire cervix. The result is that not only is the hypertrophied cervix diminished by the extent of the slough which separates, but that the healthy inflammation set up in the chronically indurated tissues gradually melt them as it were; so that often, on its subsiding, the hypertrophied cervix has regained its natural size. When this result is not obtained by the first cauterization, a second or third never fails to reduce the uterine neck to its normal dimensions. With the disappearance of the hypertrophy, also disappear the symptoms which it occasioned; the uterus returns of itself to the position which it naturally occupies in the pelvis, and the cure is really accomplished. The Vienna paste, which is composed of equal parts of quick-lime and hydrate of potassa, is applied to the uterine cervix in the following manner :—A large conical speculum must first be introduced, and the engorged cervix made to enter its orifice; or, should the cervix be too voluminous, the speculum must be firmly pressed on the part which it is ietended to cauterize, care being taken not to enclose between the rim of the speculum and the cervix a fold of the vagina. About as much of the paste as would cover a four-penny piece, must then be placed on a triangular piece of diachylon plaster, one end of which is inserted lightly in the cleft extremity of a small stick. The caustic paste then carried by means of the stick, to the cervix, and applied to the centre of the part comprised by the orifice of the speculum. With the long forceps cotton is placed carefully all round the spot on which the caustic is applied, so as to protect the neighboring parts completely; the stick is withdrawn, and the speculum is two-thirds filled with cotton or lint which is firmly pressed against the uterine neck. The speculum is then extracted, the cotton which fills it being forcibly pushed back in the vagina with the forceps, as it is pulled away, so that the vagina remains thoroughly plugged. If all this be carefully done, it is impossible for the caustic to fuse, and injure the vaginal parietes. In about fifteen or twenty minutes, the cotton or lint must be gradually withdrawn by means of a bivalve speculum, and an eschar of the size of a shilling, or rather larger, will be found where the caustic was applied. The vagina should then be washed out with a little tepid water, complete rest in bed enjoined, and emollient injections employed until the separation of the slough, which takes place from the sixth to the eighth or tenth day. This application of caustic, while it excites more inflammation than the more superficial, is rarely productive of metritis, or of inflammatory action to such an extent as to injure local abstraction of blood. The application of the actual cautery is practised by M. Jobert de Lamballe, the neighbouring parts being protected by an ivory speculum. The application is said not to be productive of much pain, and to be very successful. We need scarcely remark that such practice must be confined to the continent; English women would not submit to it, and the surgeon who should be earnest in recommending it, would soon pay the penalty by the loss of his practice.—*Ibid.*

